# Supplementary material for: Gender Differences in Factors Associated with the Total Delay in Treatment of Pulmonary Tuberculosis Patients: A Cross-Sectional Study in Selangor, Malaysia
Source: Int J Environ Res Public Health. 2022 May 21;19(10):6258. doi: 10.3390/ijerph19106258 (PMC9140698; doi:10.3390/ijerph19106258)
Supplement: Supplementary file 1 [file ijerph-19-06258-s001.zip › ijerph-1628734-supplementary.pdf]

Supplementary Table S1. List of government health clinics and hospitals included in the study

District	Hulu Selangor	Kuala Selangor	Gombak	Hulu Langat	Klang	Petaling	Sepang	Sabak Bernam	Kuala Langat
**No. of government health clinics**	6	7	10	11	11	9	4	7	10
**Government health clinics Clinics**	• Hulu Yam Bharu<br>• Rasa<br>• Serendah<br>• Sg Selisek<br>• Kalumpang<br>• Kg Soeharto	• Bestari Jaya<br>• Bukit Cherakah<br>• Jeram<br>• Kuala Selangor<br>• Sg Tengi Kanan<br>• Ijok<br>• Tg Karang	• AU2 Keramat<br>• Batu Arang<br>• Kuang<br>• Rawang<br>• Selayang Baru<br>• Taman Ehsan<br>• Ulu Klang<br>• Bt 8 Gombak<br>• Gombak Setia<br>• Sungai Buloh	• Balakong<br>• Bandar Seri Putra<br>• BB Bangi<br>• Beranang<br>• Bt 14 Cheras<br>• Bt 9 Cheras<br>• Kajang<br>• Sg Chua<br>• Ampang<br>• Semenyih<br>• Sg Sekamat	• Anika, Klang<br>• Botanik<br>• Bukit Kuda<br>• Bukit Naga<br>• Kapar<br>• Pel Klang<br>• Pulau Indah<br>• Rantau Panjang<br>• Meru<br>• Pandamaran<br>• Pulau Ketam	• Bt 13 Puchong<br>• Kelana Jaya<br>• Lembah Subang<br>• Paya Jaras<br>• Sek 7 Shah Alam<br>• Seri Kembangan<br>• Bt 14 Puchong<br>• Sek 19 Shah Alam<br>• Taman Medan	• Dengkil<br>• Salak<br>• Sepang<br>• Sg Pelek	• Bagan Terap<br>• Merbau Berdarah<br>• Sabak Bernam<br>• Sekinchan<br>• Sg Air Tawar<br>• Parit Baru<br>• Sg Besar<br>• Simpang Lima	• Jenjarom<br>• Kanchong Darat<br>• Kg Bandar<br>• Sg Lang Tengah<br>• Sijangkang<br>• Telok Panglima Garang<br>• Tg Sepat<br>• Bt 10, Kebun Baru<br>• Bukit Changgang<br>• Telok Datok
**Government hospitals**		• Hospital Tanjung Karang	• Hospital Sungai Buloh		• Hospital Tengku Ampuan Rahimah			• Hospital Tengku Ampuan Jemaah	Hospital Banting
**Sample size**	28	25	115	187	123	285	18	12	30
